# Improving CommonGround, an Online Peer Support Intervention for People Living With Long-Term Physical Health Conditions and Subthreshold Depression: Theory of Change–Informed Qualitative Study

**DOI:** 10.2196/92384

**Published:** 2026-11-03

**Authors:** Hannah Grace Jones, Ciara Regan, Grace Lavelle, Elly Aylwin-Foster, Alan Simpson, Ewan Carr, Matthew Hotopf, Vanessa Lawrence

**Affiliations:** 1 Institute of Psychiatry, Psychology and Neuroscience, Department of Psychological Medicine, King's College London, 16 De Crespigny Park, London, England, United Kingdom, +44 (0)20 784 87073; 2 Persistent Pain Research Group, Hopwood Centre for Neurobiology, South Australian Health and Medical Research Institute, Adelaide, South Australia, Australia; 3 IIMPACT in Health, School of Allied Health and Human Performanc, Adelaide University, Adelaide, South Australia, Australia; 4 Health Service and Population Research, Institute of Psychiatry, Psychology and Neuroscience, King's College London, London, England, United Kingdom; 5 CP-Life Research Centre, School of Physiotherapy, Royal College of Surgeons, Dublin, Ireland; 6 Florence Nightingale Faculty of Nursing, Midwifery and Palliative Care, King's College London, London, England, United Kingdom; 7 Department of Biostatistics and Health Informatics, King's College London, London, England, United Kingdom

**Keywords:** chronic conditions, depression, long-term physical health conditions, mental well-being, online peer support, subthreshold depression, Theory of Change

## Abstract

**Background:**

People living with long-term physical health conditions who are also experiencing subthreshold depression are at risk of developing major depression. In partnership with lived experience experts, clinicians, and software developers, we co-designed CommonGround, an online peer support and psychoeducation platform. CommonGround’s Theory of Change (ToC) was also conceptualized to outline the intermediate outcomes (and associated indicators of whether such outcomes are achieved) needed to improve the mental health of the target population.

**Objective:**

This qualitative study, informed by the ToC, aimed to examine the acceptability, usability, and intervention mechanisms of CommonGround and ultimately refine the ToC and corresponding intervention design ahead of definitive evaluation.

**Methods:**

A nested qualitative study was conducted within a remote, 12-week feasibility randomized controlled trial comparing CommonGround (intervention group) to signposting to National Health Service (NHS) web pages (control group). Thirty-nine participants were purposively sampled and interviewed within 6 weeks postintervention about their experiences of CommonGround and trial participation. The interview schedule and thematic framework analysis were rooted in a pretrial ToC while being responsive to emergent themes.

**Results:**

Evaluating the assumptions and intermediate outcomes (via their indicators) refined our understanding of how CommonGround may support mental well-being. Overall, participants viewed CommonGround as an appropriate, safe space to access self-management information and support from others with a unique shared understanding of chronic illness. However, many participants felt more time was needed for genuine social relations to form and community culture to evolve. Anonymity helped foster a sense of safety but, for some participants, limited the formation of genuine connections. Other factors contributing to low engagement included personal circumstances (eg, limited time). Key suggestions for future development included creating a mobile app that would be easier and more intuitive to access and navigate.

**Conclusions:**

CommonGround was considered an acceptable and appropriate space to access peer support, with the potential to positively impact psychological well-being by fostering shared understanding, support, and knowledge exchange. Beyond informing the future evaluation of CommonGround in a definitive trial, the refined ToC identifies the key preconditions, intermediate outcomes, and associated indicators, and assumptions that may underpin the acceptability and usability of digital peer support interventions in achieving their intended outcomes. We also demonstrate how evaluating and refining components of the ToC after the intervention had been implemented in the trial enabled a more nuanced understanding of how and why the intervention may work, for whom, under what circumstances, and informed changes to improve the co-designed intervention.

## Introduction

Living with a long-term physical health condition can have a significant effect on one’s mental health. In the United Kingdom, approximately 20% of people living with a long-term condition also have a diagnosis of major depressive disorder [1,2]. A further 20% of people are experiencing subthreshold depression [3,4], which not only impacts individuals’ physical health [5] and quality of life [6,7] but also puts them at risk of developing major depressive disorder [6,7]. The worsening of subthreshold depression into major depressive disorder has significant consequences for the individuals and their families, society, and the health care system [8-10]. Despite this known risk and calls for further research on how best to support the mental well-being of people living with long-term physical health conditions [11], there is limited targeted psychological support available for people with long-term conditions and subthreshold depression.

To address this current gap in psychological support, we initially explored the unmet psychosocial needs of those living with long-term physical health conditions and whether connecting people with similar experiences of chronic illness through peer support could help fulfill these needs [12]. Participants reported that sharing emotional support and health-related knowledge with people “like me” was invaluable. The online setting was also appreciated for offering flexible, on-demand engagement that could accommodate fluctuating mental health needs and overcome the geographical and physical barriers associated with accessing in-person peer support [12-14]. Indeed, the perceived benefits of online peer support have been widely documented among people with long-term physical health conditions. This includes evidence of new connections and reciprocal social support with people who are best placed to understand their situation [15-20], enhanced experiential knowledge, and empowerment that bolsters self-management [19,20]. Despite the potential of peer support to improve the lives of those with long-term health conditions, historically, the research agenda and the development of digital mental health interventions have largely been driven by academics and people in industry [21]. This is likely to be particularly problematic for people with long-term health conditions, who need interventions that can be easily integrated alongside existing treatment and self-management routines without adding an additional burden [22]. Putting the voices of people with lived experience at the heart of intervention design facilitates the development of interventions that align with individuals’ priorities and the challenges they face, thereby enhancing uptake and the quality of interventions [22].

In our prior work, people with long-term health conditions shared that a co-produced, safe, and credible peer support platform was much needed to address the gap in psychological support [12]. They reported that the interplay between their mental and physical health was largely ignored in their health care and that any new intervention should acknowledge the mind-body link [12]. We worked alongside lived experience experts, clinicians, and software developers to co-design CommonGround, which includes a peer support forum and psychoeducation library [23]. Our lived experience experts created the “all diagnoses welcome” ethos, arguing that as many experiences transcend diagnoses, people can provide support purely on the commonality that they are navigating life with a long-term physical health condition. To our knowledge, this is the first peer support platform that is dedicated specifically to supporting the mental health of those living with long-term health conditions and subthreshold depression.

When co-designing the intervention with key stakeholders, we also built a Theory of Change (ToC) to capture the intermediate outcomes needed for the intervention to affect change in mental well-being [12]. ToC is a participatory, theory-driven approach to intervention design and evaluation that can be incorporated into all phases of the Medical Research Council framework for complex interventions to improve our understanding of how and why an intervention works [24]. Furthermore, rooting intervention development within a co-produced ToC also increases the likelihood of the intervention being effective, sustainable, and scalable [25]. Situating intervention evaluation within a co-produced ToC that can be interrogated and refined also allows the underlying ToC to evolve in response to empirical evidence from qualitative inquiry.

In this work, we conducted a 2-arm feasibility randomized controlled trial (fRCT) to establish the feasibility and acceptability of CommonGround and the feasibility of conducting a large-scale definitive trial [26]. To allow for a richer, integrated understanding of participants’ experiences of the CommonGround platform and to strengthen the intervention and refine the underlying ToC, we conducted a nested qualitative study [27]. Located within the feasibility and piloting phase of the Medical Research Council framework for complex interventions [28], we aimed to explore participants’ experiences and perspectives to answer the following research questions:

1. How acceptable is the CommonGround intervention for people living with long-term health conditions and subthreshold depression?
2. How feasible is it for people living with long-term health conditions and subthreshold depression to use the CommonGround intervention?
3. What are the perceived benefits of CommonGround, and how can CommonGround be optimized to achieve them?

We also aimed to use the findings from this study to refine the CommonGround ToC with regard to clarifying the assumptions, intermediate outcomes, and most appropriate indicators.

## Methods

### Study Design

In this paper, we present a nested qualitative study of a mixed-methods 2-arm fRCT [26]. The CommonGround project was conducted remotely in the United Kingdom. The protocol was preregistered (ClinicalTrials.gov: NCT06222346) and is published elsewhere [26]. The CommonGround project is underpinned by a pragmatic mixed-methods research paradigm with community participation, which prioritizes what works, for whom, and in what context. The nested qualitative study presented in this paper adopts an interpretivist-constructivist approach that assumes realities are socially constructed through interactions with others within the cultural contexts in which they are situated. The pragmatic lens focuses on gathering insights that relate to actionable results and problem-solving with regard to the usability, acceptability, and refinement of the CommonGround intervention (and its ToC). This paper is reported in accordance with the SRQR (Standards for Reporting Qualitative Research) checklist (Checklist 1).

### The CommonGround Intervention

CommonGround is a web-based platform accessible via desktop and mobile devices that was open to people with diagnoses of long-term physical health condition(s). CommonGround features an anonymous community forum (peer support component) and a resource section with evidence-based self-help materials (psychoeducation component). Clinical moderators monitored the platform in accordance with our moderation, safeguarding, and privacy policies [29]. A second team, distinct from the moderation team, stimulated community discussions and responded to low-activity posts under the username of “CommonGround Engagement Team.” While the focus of the moderation team was on safety and implementing the moderation policies (eg, removal of personal information), the engagement team exclusively focused on cultivating discussions and encouraging engagement. Further details on the features of the CommonGround platform are available in the protocol [23,26] and shown in screenshots in Multimedia Appendix 1.

### Pretrial ToC

We co-created a ToC model as part of the co-production process to guide the design and evaluation of the CommonGround intervention. In this way, the pretrial ToC informed the development of the interview guides and the initial areas for exploration (eg, usability, involving focus on layout), with the ultimate aim of refining the ToC through participant feedback and perspectives. The pretrial ToC (Figure 1) includes a logical sequence of intermediate outcomes related to the acceptability of the platform (eg, “creating a space that feels safe”) and the usability of the platform (eg, “peer support platform is easy to navigate”) required to achieve our specific long-term goal of improved mental health outcomes for people living with long-term conditions and subthreshold depression [23]. Indicators were identified to explore whether each intermediate outcome was achieved, and key assumptions and gaps in knowledge were articulated for evaluation in the fRCT. By applying a ToC to articulate and refine intervention mechanisms, there is potential to strengthen complex health interventions through qualitative inquiry [30]. In this study, strengthening refers to a theory-driven, qualitative process of refining the intervention’s (ie, CommonGround) assumptions, mechanisms, and design features to improve acceptability, usability, and readiness for definitive evaluation.

Guided by the pretrial ToC (Figure 1), we explored three assumptions considered to be outside the control of the project and implementation of CommonGround but necessary for the outcomes to be achieved: (1) people with long-term conditions and subthreshold depression are willing to engage in peer support, (2) CommonGround is perceived to be personally relevant to their individual needs, and (3) participants have sufficient time and space to engage. Further assumptions in the ToC were not evaluated, relating to (1) digital literacy (we assumed participants considered themselves appropriately digitally literate by nominating themselves to take part in an online trial and self-reporting having access to the internet at baseline) and (2) access to appropriate devices and the internet (an inclusion criterion). A final assumption, (3) participants’ attitude toward how they find out about CommonGround, was considered beyond the scope of the feasibility trial.

**Figure 1.**
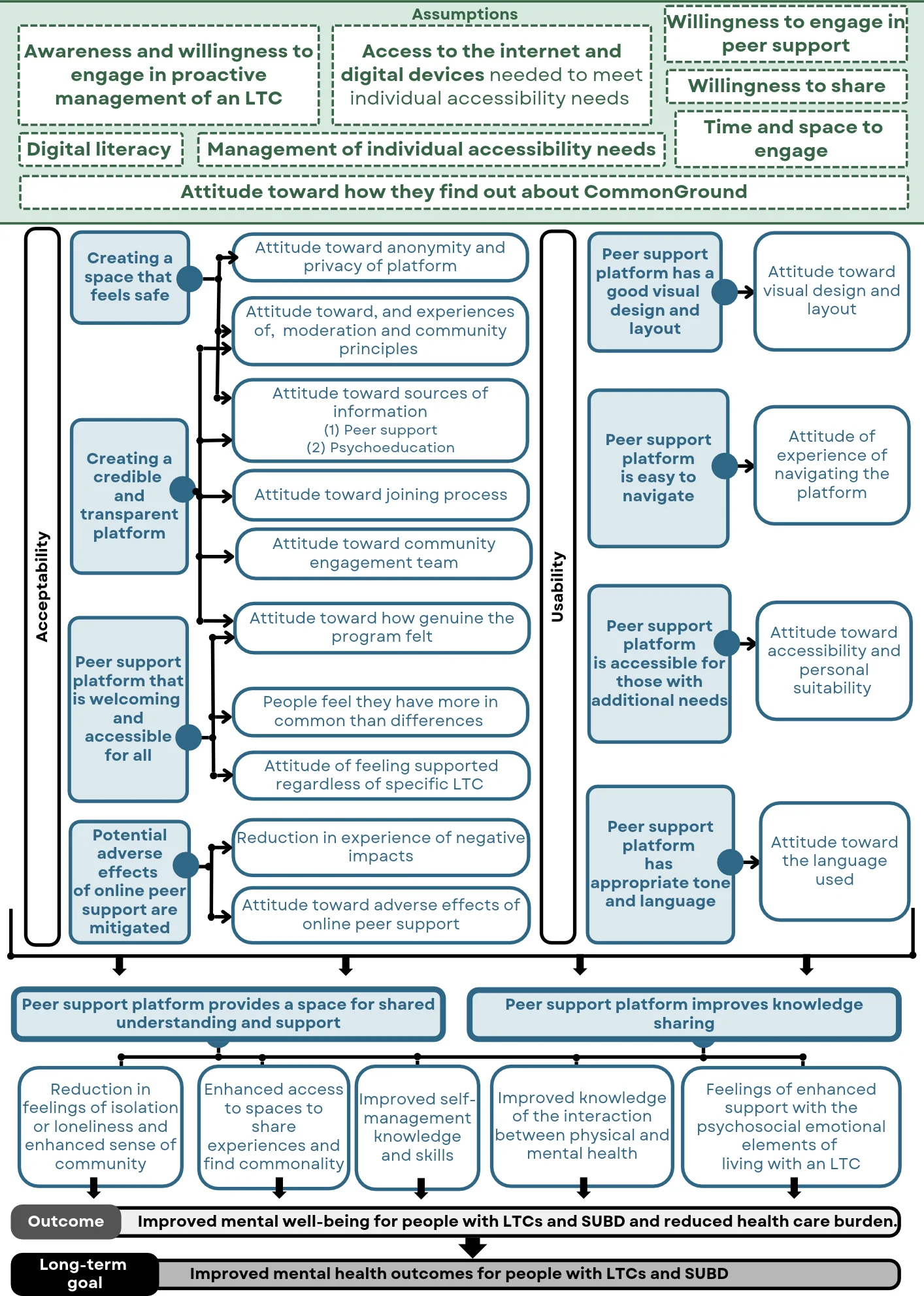
Pretrial CommonGround Theory of Change. LTC: long-term condition; SUBD: subthreshold depression.

### Sampling and Recruitment

Participants were recruited via 5 routes (Figure 2). Participants meeting the eligibility criteria (Textbox 1) were enrolled into the fRCT and given access to their randomly allocated treatment of either CommonGround (intervention) or signposting to National Health Service (NHS) Mental Health web pages (control) [31]. Post intervention, interviews were scheduled at the participants’ earliest convenience. To help the CommonGround intervention gain traction and maximize the opportunity for a community to evolve, we enrolled an additional group of participants as part of a parallel community cohort study onto the platform (for further information, please refer to protocol; [26]). This additional group scored 0 to 4 (no or low depressive symptoms) or 10 to 14 (moderate depressive symptoms) on the 8-item Patient Health Questionnaire (PHQ-8) but satisfied all other eligibility criteria. Given that symptoms of subthreshold depression may fluctuate over time, particularly in the context of living with a long-term physical health condition, these participants represented individuals with similar clinical characteristics who may move into or out of the target PHQ-8 range (5-9). Including a broader range of symptom severity also better reflects how CommonGround may be implemented in routine practice, where access may not be restricted to individuals meeting a narrow symptom threshold of 5 to 9 to effectively manage subthreshold depression and prevent the development of major depressive disorder. All participants were part of the same online community at the same time and were not distinguishable from one another. Consequently, the inclusion of the additional community cohort was unlikely to have substantially influenced participants' experiences of the platform, as interactions reflected those expected in a naturally occurring online peer support community. The flow of participants through the fRCT and community cohort study is illustrated in Figure 2.

**Figure 2.**
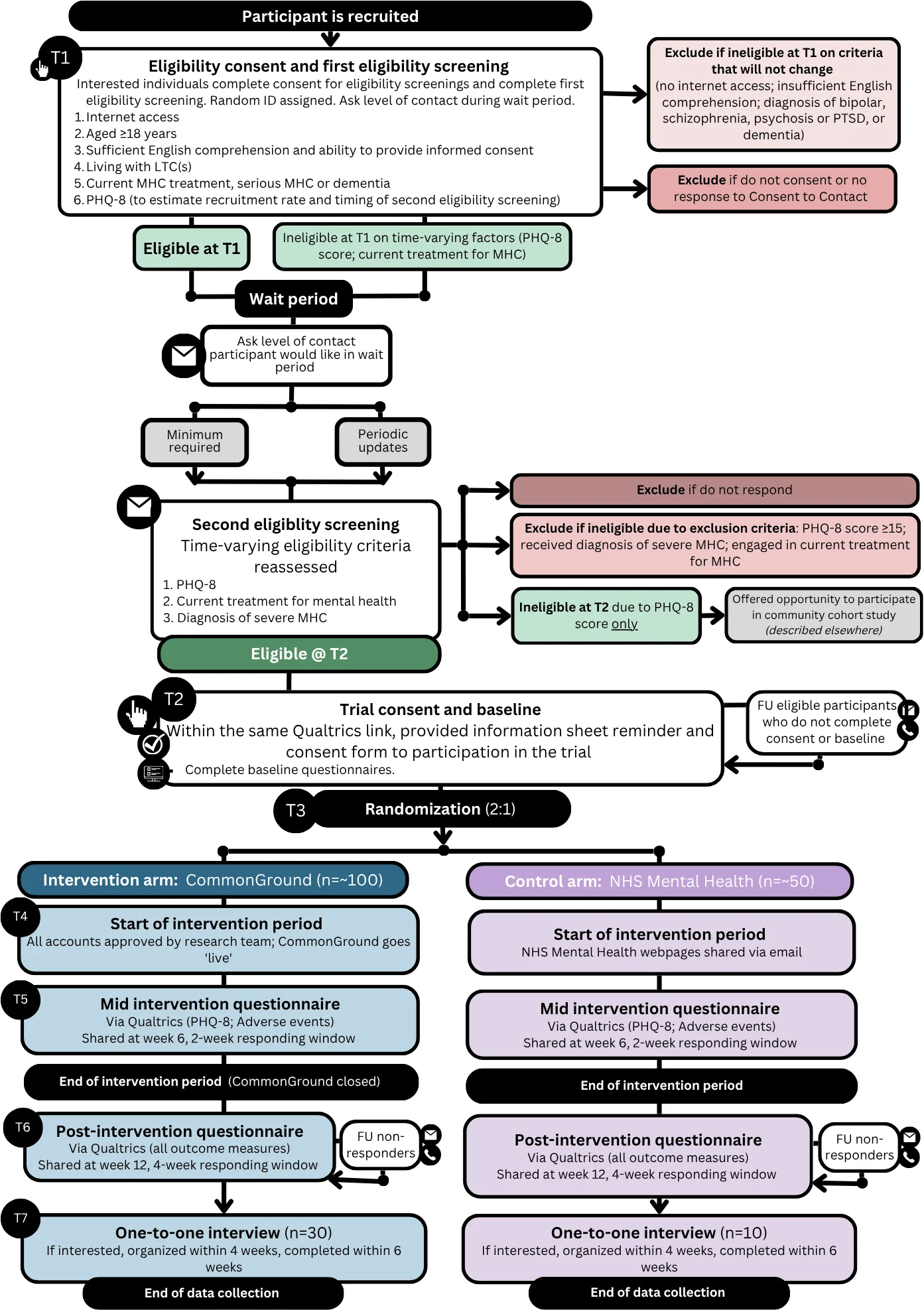
The flow of participants through the CommonGround feasibility randomized controlled trial from recruitment to end of data collection. FU: Follow-up; ID: Identification number; LTC: Long-term health condition; NHS: National Health Service; MHC: Mental health condition; PHQ-8: Patient Health Questionnaire-8; PTSD: Post-traumatic stress disorder

Textbox 1. **Eligibility criteria for the feasibility randomized controlled trial (fRCT) and community cohort samples.**

fRCT

- Inclusion criteria

-  Currently living with 1 or more long-term conditions. A long-term condition was defined as a diagnosed physical medical condition with a duration of ≥6 months that demonstrates recurrence or deterioration and is typically associated with a poor prognosis [32]. There were no restrictions on the type of long-term condition.
-  Probable subthreshold depression, defined as scoring 5-9 on the 8-item Patient Health Questionnaire (PHQ-8).
-  Aged ≥18 years.
-  Have access to the internet (via phone, desktop, or laptop at any location, including at home, at the workplace, or free Wi-Fi in public spaces).
-  Have sufficient English proficiency to engage with the platform.
-  Ability to give informed consent (ie, no known circumstances/conditions that may affect the capacity to consent).

- Exclusion criteria

-  Has ever received a clinical diagnosis of severe mental illness of bipolar disorder, psychosis, posttraumatic stress disorder (PTSD), and/or schizophrenia.
-  Has ever received a clinical diagnosis of dementia.

Community cohort

- Inclusion criteria

-  Currently living with one or more long-term conditions. A long-term condition was defined as a diagnosed physical medical condition with a duration of ≥6 months that demonstrates recurrence or deterioration and is typically associated with a poor prognosis [31]. There were no restrictions on the type of long-term condition.
-  Scoring 0-4 or 10-14 on the PHQ-8.
-  Aged ≥18 years.
-  Have access to the internet (via phone, desktop, or laptop at any location, including at home, at the workplace, or free Wi-Fi in public spaces).
-  Have sufficient English proficiency to engage with the platform.
-  Ability to give informed consent (ie, no known circumstances or conditions that may affect the capacity to consent).

- Exclusion criteria

-  Has ever received a clinical diagnosis of severe mental illness of bipolar disorder, psychosis, posttraumatic stress disorder, and/or schizophrenia.
-  Has ever received a clinical diagnosis of dementia.

### Qualitative Data Collection

After the intervention period (12 wk), a subsample of participants who had accessed CommonGround (ie, intervention group and community cohort) were purposively recruited based on their long-term conditions, age, gender, ethnicity, and engagement level to explore a range of perspectives (Table 1). We aimed to conduct approximately 40 interviews, as this was estimated to afford sufficient information power, given our broad research questions, heterogeneity in the participant group, and across-case approach to analysis [33]. Each participant was sent an initial email, followed by a phone call to discuss the interview and answer any queries. Participants were given a full explanation about the interview and allowed time to consider their participation before providing informed consent. All participants were free to withdraw at any time or to decline to answer a question without having to give a reason.

**Table 1.** The different engagement activity groups that were purposively sampled for interview and example topic guide questions^a^.

Engagement activity group	Example question from topic guide tailored to style of engagement
Never joined: Never registered their account or logged in	*Why did you choose not to create an account?*
Never logged in**:** Created an account but never logged in	*What would you say the main reason(s) for not using CommonGround were?*
Readers**:** Logged in but never commented, reacted, or posted	*The peer support platform included an online discussion forum called the community page. You could create posts, ask questions, share your experiences, react, and comment on other people’s posts. What do you think about this feature? Did you use it? If not, why not?*
Acknowledgers**:** Logged in and commented and/or reacted, but did not post	*How did you use the community page? What did you think of the posts and comments you looked at or used?*
Content creators**:** Logged in and created posts	*What motivated you to create post(s)?*

a Number of logins within each category varied from rarely (eg, once a month) to frequently (eg, multiple times a week).

The individual interviews were conducted online via Microsoft Teams by one member of the research team (HGJ, GL, or CR). Interviewers debriefed between interviews to review the topic guides. The topic guides (Multimedia Appendix 2) were used flexibly, taking into account participants’ different levels of engagement with CommonGround to further explore specific experiences. For instance, greater emphasis was placed on understanding reasons for nonadherence among those who never logged in versus motivations for posting, and the sense of community among frequent users. The general aim of the interviews was to explore participants’ experiences of the CommonGround intervention, examining its acceptability, usability, and perceived benefits and limitations in relation to the intermediate outcomes and assumptions identified in the pretrial ToC (Figure 1). The interviews lasted between 30 and 90 minutes and were audio recorded before being transcribed verbatim (by Way with Words) and anonymized.

### Qualitative Analysis

Thematic framework analysis was used to facilitate analysis within and between individual cases and groups of participants [34,35]. The thematic framework drew on *a priori* indicators identified in the pretrial ToC, while being responsive to emergent themes. Our interpretivist-constructivist approach recognized that participants’ experiences and interpretations of the peer interactions, psychoeducation, and other aspects of CommonGround were subjective and shaped by their individual social, personal, and contextual circumstances. In particular, the social experiences that took place on the CommonGround platform were also considered to be socially co-constructed between participants. The pragmatic lens also brought focus to identifying areas to enhance or further develop CommonGround in the future.

All analyses were conducted in NVivo (version 14; released in 2023; Lumivero) by 2 coders (HGJ and CR). After independently coding 5 interviews, the coders discussed alternative viewpoints and reviewed the coding framework, adding, collapsing, and refining codes. This process enhanced analytical rigor by incorporating multiple perspectives into the interpretation of the data and helped ensure that the emerging themes remained grounded in participants’ accounts. The Research Advisory Group (RAG) then reviewed the coding framework, identifying missed topics and refining the interpretations of codes. Both coders then recoded 1 interview using the updated coding framework to confirm consensus on its application. Once all transcripts were analyzed, data were charted to map the dataset as a whole, and the RAG and the wider research team were consulted to offer their interpretations to create the posttrial ToC.

### Ethical Considerations

The study was conducted remotely in the United Kingdom by King’s College London and received ethical approval from the South Central—Oxford C Research Ethics Committee (IRAS ID 328175). This project is sponsored and organized by King’s College London. Informed consent was obtained from all participants at two stages: (1) at T_1_ for completion of the 2 eligibility screenings, and, if found to be eligible for the trial, (2) at T_2_ to participate in the trial (Figure 2). For participants invited to take part in an interview, the study and consent procedures were reviewed again before they agreed to participate. Before each interview commenced, participants were reminded of their right to withdraw at any time. Data were anonymized and deidentified during transcription. Participants were reimbursed with a GBP £20 (US $26.72) voucher as a token of appreciation for their time.

### Patient and Public Involvement

Patient and public involvement was integral throughout the CommonGround project. The CommonGround intervention was co-produced with people with lived experience of long-term physical health conditions who had experience of and interest in mental health [23]. The study protocol was also shaped by our co-investigator with lived experience of multiple long-term conditions (EA-F) and the RAG, who continued to meet and advise on the conduct of the fRCT and nested qualitative study. Our RAG involved 10 members (women: n=6, 60%; and men: n=4, 40%, aged between 25 and 73 y), living with various long-term physical health conditions. Six members were living with multiple long-term conditions, and 5 had comorbid mental health diagnoses. Our RAG co-produced the interview schedules and informed the coding framework to ensure that it was grounded in their lived experiences (reimbursed GBP £20 [US $26.72] per hour). Our co-investigator with lived experience attended meetings throughout the planning, conduct, and analysis of the trial and this study to provide iterative feedback and also chaired the RAG meetings.

## Results

### Participants

A total of 57 participants were invited to interview, of whom 39 (68%) agreed to be interviewed, 4 (7%) declined, and 14 (25%) did not respond to the email or telephone invitation (response rate to invitation: 43/57, 75%). A total of 39 participants who had been provided access to the CommonGround intervention were interviewed (29 fRCT participants with long-term conditions and subthreshold depression at baseline, 10 community engagement participants with long-term conditions and a PHQ8 score of either 0‐4 or 10‐14 at baseline). This included 21 (54%) women and 18 (46%) men representing a total of 31 distinct long-term conditions (Table 2).

**Table 2.** Participant demographics of those interviewed who had access to the CommonGround platform.

Category	Overall (N=39)
Age (years)	
Mean (SD)	53.2 (13.7)
Gender, n (%)	
Female, including transgender females	21 (53.8)
Male, including transgender males	18 (46.2)
Ethnicity, n (%)	
Asian or Asian British	—^a^
Black, Black British, Caribbean, or African	—^a^
Other ethnic group	—^a^
Prefer not to say	—^a^
White	31 (79.5)
Mixed or multiple ethnic groups	—^a^
Engagement level with CommonGround, n (%)	
Never joined	6 (15.4)
Never logged in	5 (12.8)
Reader	5 (12.8)
Acknowledger	7 (17.9)
Content creator	16 (41.1)
Total number of LTCs^b^ represented, n	31

a Fewer than 5 participants, removed to protect anonymity.

b LTC: long-term condition.

### Updates to the ToC and Refinements to CommonGround

The assumptions and the intermediate outcomes were updated and refined based on the insights from the participants to create the posttrial ToC (illustrated in Figure 3). Key changes included (1) collapsing indicators together where they were found to reflect a broader concept (eg, attitude toward the engagement team, attitude and experiences of moderation, attitude toward the joining process, and attitude toward anonymity were all combined into the indicator: attitude toward anonymity, privacy, and governance on the platform), (2) refining the assumptions (eg, a new assumption of “attitude toward personal suitability” created to capture new insights alongside pretrial assumptions of willingness to be proactive and management of complex needs); and (3) the removal of indicators that were captured by other indicators.

**Figure 3.**
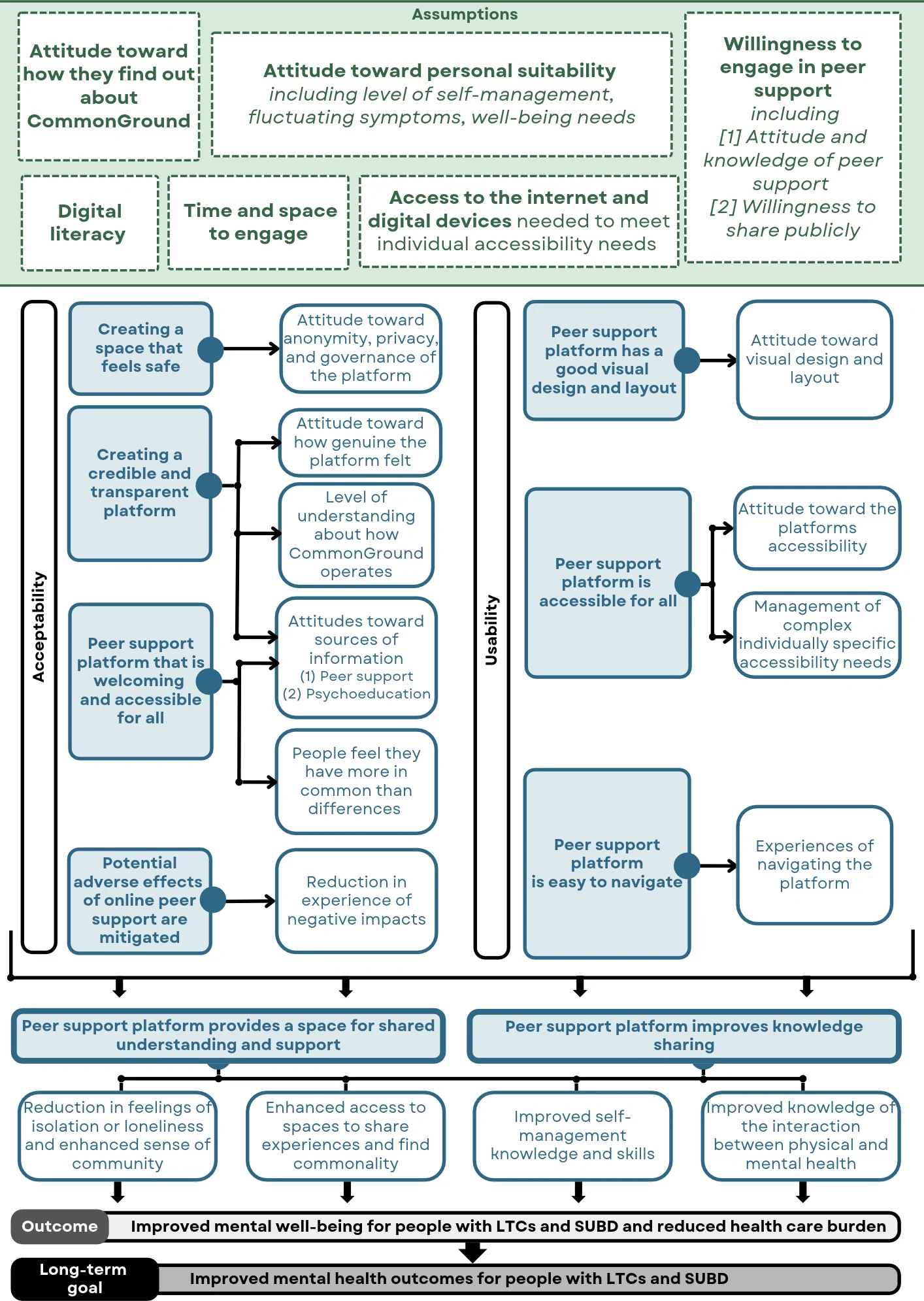
The posttrial Theory of Change. LTC: long-term condition; SUBD: subthreshold depression.

### Findings: Evidence for the ToC Assumptions and Intermediate Outcomes

#### Overview

The findings are organized in accordance with the posttrial ToC (Figure 3), demonstrating how participants’ experiences confirmed, refined, or challenged the pretrial assumptions and intermediate outcomes. We first present data relating to the 3 assumptions that were evaluated in this feasibility study, followed by the intermediate outcomes concerning acceptability, usability, shared understanding, and support, and knowledge sharing.

#### Assumption 1: People With Long-Term Conditions and Subthreshold Depression Are Willing to Engage in Peer Support

For any peer support platform to work, its members need to be willing to engage and share with others. The nature of an individual’s views about peer support affected their willingness to share, alongside other factors such as personality or communication styles. Most participants communicated a strong understanding of peer support and considered it highly valuable, with many having used condition-specific groups before. Peer support was described as an opportunity to exchange emotional support, knowledge, and advice. The mutual understanding that connecting with peers affords was appreciated, reducing the need to provide extensive explanations or justifications of their experiences. For some, the informal online forum format and 24/7 availability were key benefits:

*So, I think peer support, for me, is just being able to speak to people who have had the same or very similar experiences, because there’s something about feeling heard and believed that is really helpful.*

[Participant 29, content creator]

*Health professionals who, for them, it’s their nine to five. People with certain medical conditions, it is your 24/7, so, naturally, you'll be an expert in the area, more than medical professionals will be.*

[Participant 9, content creator]

A few participants expressed concerns about the concept of peer support that impacted their willingness to engage: some worried about the accuracy of the health-related information shared by peers, while others found the tone of some peer discussions to be overly negative and reinforcing of the distressing aspects of chronic illness. A general concern about online peer spaces was the potential for negative or hostile interactions. Those with minimal or no engagement with the CommonGround platform were most likely to express skepticism about potential benefits and concerns about negative experiences:

*I find [social media platform participant used prior to CommonGround] is very fraudulent. There’s lots of people on there who are frauds, basically, to make money, or pretend they have something, or they don't have something, so I find it very difficult. I don't trust it.*

[Participant 15, reader]

#### Assumption 2: CommonGround Is Perceived to Be Personally Relevant to Their Individual Needs

A participant’s physical and/or psychological wellness needs and other personal circumstances strongly influenced their engagement with CommonGround. Some participants shared that in the past, peer support had not aligned with their needs, either due to accessibility barriers, such as the rarity of their condition, or because the available groups did not reflect their experiences. One challenge associated with engaging with existing peer support groups was finding people “like me,” which motivated some participants to join CommonGround, hoping to connect with others with more similar experiences due to the “all diagnoses welcome” ethos, which could facilitate connections through trans-diagnoses commonalities:

*It’s always a bit different, actually, because I have Young Onset Parkinson’s, and the vast majority of people are much older. So, I’m seeming like a bit of an odd one out in those situations.*

[Participant 17, acknowledger]

The stage of diagnosis also influenced participants’ perceptions of the suitability of peer support: many believed peer support was most beneficial in the early stages of diagnosis when they are adjusting and seeking guidance. As most participants had passed this stage, they often felt less of a need for additional support, having already developed coping mechanisms and a self-management routine. However, several participants acknowledged the instability of long-term conditions and mental health, recognizing that while they did not need support at that time, having access to it for future difficulties was reassuring. Others valued peer support not for seeking help but for maintaining connections, hearing others’ experiences, or “giving back” by offering the type of support that they needed when navigating their own diagnoses:

*As a newly unwell person, those forums were literally a lifesaver, and whilst I was stuck at home and housebound or missing 70% of school, it was my entire teen social life. So, I see the importance of peer support, and it’s something that I've been involved in since that point... by being in these communities, you find edges of your tribe, who understand, and it can actually focus your diagnosis journey.*

[Participant 6, acknowledger]

*I don't feel that I have any mental health issues now, but because I went through that all those years ago, I felt I'm in a position where I may be able to help someone, just even speaking to them.*

[Participant 11, content creator]

#### Assumption 3: Participants Have Sufficient Time and Space to Engage

To experience the benefits of peer support, people must have the time and space to engage with it. Many participants did not feel like they had sufficient time for a variety of reasons. For some participants, other priorities took precedence during the trial, while for others, engaging felt unachievable or was an additional burden when experiencing a low point with their mental or physical health. Many shared that during busy times, they were most likely to log in following the weekly email sharing community updates, which served as a friendly reminder that CommonGround was there:

*It was running at a time when I was really, really busy, for various reasons, and so I didn't have a lot of free time. I might have looked a bit more often if I'd had more free time, but it was a particularly hectic time.*

[Participant 14, never logged in]

### Acceptability: Is CommonGround Acceptable?

#### Overview

Whether people with long-term health conditions and subthreshold depression considered the CommonGround to be an acceptable intervention that could support their mental well-being centered around four intermediate outcomes (Figure 3): (1) creating a space that feels safe, (2) creating a credible and transparent platform, (3) a peer support platform that is accessible and welcoming for all, and (4) mitigating the potential adverse effects of online peer support.

#### Creating a Space That Feels Safe (ToC Intermediate Outcome)

##### Attitudes Toward Anonymity, Privacy, and Governance on CommonGround (ToC Indicator)

Most participants agreed that the anonymity of CommonGround fostered a sense of safety by helping them to feel less exposed in the community. Anonymity supported them in sharing their experiences comfortably without fear of judgment or trolling. Pseudonyms and avatars helped them to cast off preconceptions of others and to speak openly about their condition in a way that they otherwise would not if sharing their real identities:

*It felt like a nice little bubble that you could be a bit more vulnerable in, online. And also, I think the anonymity of it, as well, lent to that ability to be vulnerable, because the old Oscar Wilde quote, you give a man a mask, he'll tell you the truth.*

[Participant 10, content creator]

However, some felt that enforced anonymity made the space feel very formal and inhibited the development of the deep personal connections that they considered necessary for peer support to flourish. Others recognized the benefits and drawbacks of anonymity and questioned whether there should be personal choice over whether they are identifiable to their peers.

The moderation team was considered to play a key role in creating a safe environment. Many commented that their mere presence was likely to discourage inappropriate behavior. Participants felt that their privacy was well maintained, with little concern about providing personal data to sign up, as the information requested felt appropriate and they trusted that it would be stored securely.

### Creating a Transparent and Credible Platform (ToC Intermediate Outcome)

#### Level of Understanding About How CommonGround Operates (ToC Indicator)

Overall, participants had a good understanding of CommonGround. Most participants understood the purpose of the platform and how it would be run, expressing awareness of the need for community principles, moderation, and safeguarding policies to facilitate consistent governance. Some had limited knowledge of the specific content of the policies but assumed they were reasonable, as no “red flags” had stood out when they had skimmed through this information. Although considered clear and comprehensive, participants often felt these policies were long and desired shorter, more digestible versions. The signposting for crisis support was clear, and participants appreciated the transparency that one-to-one crisis support was beyond the remit of CommonGround. Many participants were either unaware of the existence of separate engagement and moderation teams, often perceiving them as one overall team or having limited understanding of their differences in their respective functions. For many participants, however, this was not an issue, and they appreciated the overall roles that these teams were playing (ie, to keep people safe and to stimulate discussions).

#### Attitudes Toward How Genuine the Platform Felt (ToC Indicator)

Participants considered the behavior of these moderation and engagement teams to be highly pertinent to how genuine the platform felt. Participants judged the level of moderation as appropriate as the blend-into-the-background approach balanced safety without creating feelings of being watched or censored:

*I think the moderation in the background is better, because, otherwise, some people will think, oh, big brother is stepping in and doing things. They're there to protect you.*

[Participant 30, content creator]

The activity of the engagement team (who shared discussion topics and lived experience posts on the community page) was seen by the majority as helpful for prompting discussions and essential for ensuring that all forum posts received responses in the formative stages of the community:

*It felt quite nice to know that your posts, even if other people on the group hadn’t, it could be a few days before you might get a response to something, but quite often, one of the engagement team had come in and said, basically, they see you and they hear you, and that was quite nice. So, it made it feel safe. It made it feel quite inviting and nice.*

[Participant 29, content creator]

The inclusion of someone with lived experience on the engagement team was felt to increase the credibility of posts from the engagement team. However, many users also wished that the engagement team would post less frequently, as posting too frequently risked interfering with the natural flow of peer conversations and undermining the authenticity of the community:

*There were a few early conversations where somebody broached a subject. And a couple of contributors then replied to it. And then I think the engagement team came along and said, good conversation, everybody. And that was like pouring a bucket of cold water on it, I felt.*

[Participant 24, content creator]

#### Attitude Toward the Sources of Psychoeducation (Self-Help Information on the “Resources” Page; ToC Indicator)

The CommonGround platform tended to be viewed as a credible, reliable, and trustworthy source, yet some participants preferred to access information directly from medical professionals or from familiar sources who promised more specific and up-to-date information. Some had previously accessed the information elsewhere but recognized that it may be useful for others, particularly those who had received their diagnosis more recently:

*I glanced at them, to be honest. I didn't really bother very much with that at all. And I think that’s probably, as I say, because some of it is perhaps information that I already knew.*

[Participant 10, content creator]

### Creating a Peer Support Platform That Is Welcoming and Accessible for All (ToC Intermediate Outcome)

#### Whether People Feel They Have More in Common Than Differences (ToC Indicator)

The majority of participants identified value in CommonGround being a shared forum for people with different conditions and the types of conversations that emerged in this space. A strong sense of commonality existed in their shared experience of navigating life with a long-term condition, which transcended individual diagnoses and generated the exchange of useful information and support that felt unique to CommonGround:

*I think that’s good [that CommonGround is all diagnoses welcome], because it’s not necessarily about the condition that you've got. It’s about the experience that you have, living with that, and regardless of what condition it is a lot of us have the same difficulties, or we can celebrate the same kind of things.*

[Participant 42, reader]

For some, the “all diagnoses” model of peer support differed from the condition-specific peer support they had previously experienced and took some time to adjust to. At times, the mixed-diagnosis nature of the community stimulated conversations that were either too generic or lacked depth or made participants feel compelled to explain their diagnoses to develop deeper connections, which became cumbersome. Those with the lowest levels of engagement complained that they did not have time to sift through condition-specific content that felt unrelatable.

It was suggested that for the CommonGround community to work, people should focus less on explaining their conditions and more on topics that transcend diagnoses, such as adapting one’s lifestyle and coping mechanisms. While most felt that anonymity was important for safety and self-expression, some suggested that not having background information about the people on the platform made it harder to establish commonality and, consequently, harder to develop a sense of community. Therefore, while many appreciated the all-diagnoses ethos, there was a clear view that some changes were needed for the community to form properly and that more time or reduced anonymity might facilitate this. Within the mixed-diagnosis community, some participants purposefully sought out or created condition-specific threads. They valued the sense of connection they gained in these interactions and felt reassured about their experiences:

*As soon as I mentioned I had Crohn’s, there was loads of people messaging you back straight away, saying how long they had it for, and what they do there to look after themselves, so it was really helpful.*

[Participant 26, content creator]

However, for others, these condition-specific threads felt unrelatable, and they lacked the confidence to contribute. In general, participants suggested that if people wanted condition-specific support, it should be accessed elsewhere.

#### Attitude Toward the Information Shared on CommonGround (ToC Indicator)

The majority of participants valued having a space where the content was exclusively created by people with long-term conditions, which felt safer and more understanding environment than other online spaces. There were split opinions on the appropriateness of discussing medical topics on CommonGround, and some participants were concerned with the pessimistic tone of some peer discussions. The relatively low levels of engagement and the community’s small size and early stage of formation were cited as factors that reduced the relevance of the posts during the trial period, but would likely evolve over time although these factors were expected to change over time:

*A lot of the times when I went on, if there were new posts, they were very visceral energy. I really felt like this is really like bringing me down, rather than making me feel better*.

[Participant 12, content creator]

### Views on Whether the Potential Adverse Effects of Online Peer Support Are Mitigated (ToC Intermediate Outcome)

#### Attitude Toward the Governance and Approach to Reduce the Experience of Negative Impacts on CommonGround (ToC Indicator)

The majority of participants did not interact with the moderators nor see anything that needed moderation. This was considered a positive, as while many felt that moderation was essential, they suggested it should blend into the background

*I didn't come across any points where I could see that they'd been involved, moderated something I'd written, or something that somebody else had written. But it was nice to know that they were there. That it was being moderated. And that’s invaluable for me, I think.*

[Participant 24, content creator]

As mentioned, the simple presence of the CommonGround moderation team contributed to feelings of safety. One participant who had their post moderated shared their desire for more information on why the moderation was necessary, calling for increased communication from the moderators regarding their specific situation to supplement the comprehensive policies.

### Usability: How Usable Is CommonGround?

Whether participants were able to use the CommonGround intervention as desired was centered around three intermediate outcomes (Figure 3): (1) providing a peer support platform with good visual design, (2) providing a peer support platform that is easy to navigate, and (3) providing a peer support platform that is accessible for all.

#### Providing a Platform That Has Good Visual Design (ToC Intermediate Outcome): Attitudes Toward the Visuals, Layout, and Uniformity of the Platform (ToC Indicator)

Most participants liked the visuals, layout, and color scheme of CommonGround, which appeared professional, warm, and inviting. One participant also appreciated the lack of advertisements and visual distractions on the platform in comparison to other online spaces:

*I really liked the layout, and the imagery was all very friendly and welcoming. So, yes, a big fan*

[Participant 30, Content creator]

#### Providing a Peer Support Platform That Is Accessible for All (ToC Intermediate Outcome): Attitudes Toward the Accessibility of the Platform (ToC Indicator)

##### Overview

Many participants found that CommonGround’s features supported engagement and accessibility, fulfilling their expectations of what a peer forum should offer and were frequently used. The unique reaction buttons (eg, “I hear you”; “I support you”) felt different from those on other online spaces and more aligned with the type of reactions that they wanted to receive when sharing lived experience. Many found the reaction buttons to be an easy way to respond to posts or show support, particularly if they were unsure of what to comment. However, on occasion, participants suggested that these reaction buttons could discourage written responses from peers:

*One of the things I really liked about the platform is that people could... They didn't have to comment as such. They could just click. And I think the, I hear you, comment, or, I support you, is really invaluable. It’s a real boost to people*.

[Participant 10, content creator]

Many saw the potential usefulness of being able to curate their own library of saved posts and resources if the community was larger and there was more content. However, the My Garden feature, which allowed participants to build their personal library, was often not used, with some being unaware that the page existed. A few mentioned that if CommonGround were a mobile app with push notifications, it would improve accessibility and usability and help overcome challenges experienced in the web-based version. In general, the registration process was considered straightforward; however, a few participants who had never logged in reported issues with creating acceptable passwords or other technical issues that created difficulties with registration. Throughout their interviews, many participants suggested areas for improving CommonGround, which are outlined in Table S1 in Multimedia Appendix 3.

##### Perceived Management of Individuals' Complex Accessibility Needs (ToC Indicator)

Few participants raised complex accessibility needs. Some noted physical accessibility issues, including problems using a computer due to tremors or an inability to sit at a desk for an extended period. Suggested solutions included incorporating a voice-to-text feature to avoid typing and autogenerated responses for responding to peers’ posts. A small number of participants highlighted the need for improved accessibility features on the types of support that (eg, dark mode) for those who are color blind or visually impaired.

### Providing a Platform That Has Intuitive Navigation (ToC Intermediate Outcome): Experiences of Navigating the Platform (ToC Indicator)

Participants recognized that the 2-factor authentication (2FA) step at login (occurring once in a 24-h period) was needed for security and data protection; however, many expressed frustrations and disliked this feature overall as it made logging in more difficult than needed. Many suggested that having a mobile app of the platform could reduce the need for 2FA:

*I've got to go through multi-factor authentication and faff, faff, faff. So, it just seemed overly secure. Yes, but if it hadn’t have been, I would have quite happily been dipping in and out a lot more frequently.*

[Participant 10, acknowledger]

Many participants found CommonGround simple to use, but some were unaware of the additional pages they could access, which limited their engagement with these pages. Some participants also found the community forum threads difficult to follow, especially when conversations became long or fragmented, indicating a need for clearer thread organization or notifications.

### What Are the Benefits of CommonGround and How Might They Be Optimized?

#### Overview

To evaluate our ToC relating to the different ways in which CommonGround may support the mental well-being of people with long-term conditions and subthreshold depression, two key pathways were considered (intermediate outcomes; Figure 3): (1) CommonGround provides a space for shared understanding and support and (2) CommonGround improves knowledge sharing.

#### Peer Support Platform Provides a Space for Shared Understanding and Support (ToC Intermediate Outcome)

##### Does CommonGround Enhance Access to Spaces to Share Experiences and Sense of Commonality? (ToC Indicator)

Participants had mixed experiences about whether CommonGround enhanced their ability to share experiences and find a sense of commonality. Some explained that without a pre-established mutual understanding, which could be assumed within a condition-specific space, they were hesitant to comment on others’ experiences or offer advice. This sometimes placed constraints on the types of support that were offered, as participants were hesitant to say the “wrong thing”:

*It [the all-diagnosis-welcome ethos] can mean that some things are more relevant than others. And also, sometimes, things, like responses, can be a bit generic or a bit general and maybe not as specifically relevant.*

[Participant 26, content creator]

Some participants sought out connections with people with the same condition but struggled to find them, which affected their motivation to share. Yet despite this, most saw the potential for the platform to facilitate shared experiences if it were to continue to grow: the short time frame and the low level of peer-generated content limited the sense of community that could form. Others placed value on the diversity of experiences that were shared across a range of conditions, identifying this as enriching, rather than reducing, their sense of connection. These participants described feeling understood, supported, and validated by others. Seeing other participants receive supportive responses also increased their confidence in posting:

*I think it was very good that you have a mix. Yes, absolutely, because the condition doesn't define you. I've got one condition. Somebody else has got another condition. How it impacts you physically, emotionally, mentally can be exactly the same, or you can have very similar experiences for different reasons. How it affects your family life. How it affects your relationship, either with your partner or with friends. How it affects you, your ability to go out of the house, some days, even. It doesn't matter what the condition is. It can all have a very similar effect on you, so it gives you that broader spectrum.*

[Participant 10, content creator]

As discussed, anonymity played a complex role in participants’ experiences of CommonGround. In relation to creating a space to share and find commonality, for some, the anonymity created a lack of trust among peers and reduced their willingness to post. For others, it engendered a feeling of freedom from judgment that allowed for greater vulnerability in the online space.

##### Does CommonGround Reduce Feelings of Loneliness and Enhance a Sense of Community? (ToC Indicator)

A positive sense of community was experienced by many, despite their awareness that the community had not yet reached its full potential. This was driven by the kindness of their peers, “authentic” expressions of care and support, and the noncommercialized nature of CommonGround. Overall, CommonGround was considered a safe, judgment-free space, which supported those wishing to share lived experience. Simply reading community comments and receiving replies to their own posts helped reduce loneliness:

*It was really good to hear other people’s posts because, as I said before, it’s really good to hear how other people feel and what they go through. And it makes you feel, yes, this is exactly how I feel, so it’s normal to feel that way. It’s not all in your head. It’s real. It’s things that do happen and other people are feeling the same way.*

[Participant 16, acknowledger]

For some participants, anonymity limited the depth of connection and inhibited trust-building, thereby limiting their personal experiences of community. But they often recognized how user avatars and “following” members did help to foster feelings of familiarity in the anonymous environment and witnessed others experiencing community membership. Many who felt like an “outsider” also attributed this feeling to their own lack of engagement with the platform, which was sometimes related to their difficulty in connecting with the forum content or lack of time. It was clear that more time was needed for the community to form and to reduce the occurrence of slow and fragmented conversations that lacked natural rhythm or discouraged members from sharing their own experiences, fearing that their posts would go unanswered:

*I would think, well, if I post something, no one’s probably going to reply anyway, so I may as well go and post it somewhere else.*

[Participant 42, reader]

Additionally, some participants felt that while the engagement team posts helped to stimulate conversation, the high frequency could inhibit organic, peer-led conversation. The short duration of the intervention also limited opportunities for deeper relationships to form, and some participants noted that interactions often felt transactional or impersonal. It was suggested that with more time, deeper conversations and stronger connections might have emerged, and a stronger sense of community may have evolved.

### Peer Support Platform Improves Knowledge Sharing (ToC Intermediate Outcome)

#### Does CommonGround Improve Self-Management Knowledge and Skills? (ToC Indicator)

There was a consensus that the community forum was more beneficial than the resources section for learning self-management strategies, illustrating a preference for the peer-generated content. The resources section was not widely used, and some participants were unaware it existed. However, a few participants found the resources beneficial, noting that they regularly revisited them for self-management tips. Others, while not actively using them, valued knowing the information was available if needed.

#### Does CommonGround Improve Knowledge on the Interaction Between Physical and Mental Health? (ToC Indicator)

Many participants recognized the mind-body connection when they spoke about their health, often suggesting that it was an intrinsic part of the experience of living with a long-term condition. Some participants valued how CommonGround acknowledged the complexity of living with a long-term condition with both its physical and mental health dimensions. As the interaction between physical and mental health was not explicitly addressed in the topic guide, our interpretations beyond this are limited.

### Overall Synthesis of Qualitative Findings Within the ToC

#### Were the Indicators, Assumptions, and Immediate Outcomes of the ToC Satisfied?

Consistent with the ToC approach, the qualitative findings were synthesized against the refined posttrial ToC to identify which assumptions and intermediate outcomes were supported, which required modification, and which were not substantiated. Table 3 summarizes the indicators of the posttrial ToC and whether each indicator (and therefore the corresponding assumption and intermediate outcome) was satisfied. Overall, 8 of the assumptions and intermediate outcomes were judged to be well-founded or required only minor adaptations, and 10 of the assumptions and indicators were not met (Table 3).

**Table 3.** The assumptions and indicators evaluated and the outcome.

Area of ToC^a^ and intermediate outcome	Indicator evaluated	Was the indicator satisfied?
Usability		
Accessible for all	What is people’s attitude towards accessibility of the platform? Does the platform account for individuals’ complex accessibility needs?	Yes, mainly positive with some areas of improvement Unclear, need further evaluation
Good navigation and design	Do people like the visuals, layout, and uniformity? Can people navigate the platform?	Yes, majority liked Mix, frustration with some elements (2fa); once they were logged in most found it easy to navigate, but some did not
Acceptability		
Place that feels safe	How do users feel about the anonymity and privacy on the platform?	Yes, privacy and anonymity were well maintained
Credibility and transparency	Did participants understand how CommonGround worked? Did CommonGround feel authentic to users? Did users trust the psychosocial information and resources?	Yes, generally good understanding Yes, most participants felt that the platform was genuine, though a minority felt the engagement team’s frequent posts reduced its authenticity. Yes, most felt that its reliable source being on the research site – no one explicitly mentioned that it seemed untrustworthy
Welcoming and accessible for all	Do users feel like they have more in common than separated them? How comfortable did people feel interacting with the community page?	Mixed, many did, others did not – used to condition-specific spaces Yes, people felt comfortable
Potential adverse effects are mitigated	How well did CommonGround mitigate the potential adverse effects of online peer support?	Yes, minimal adverse effects observed, perceived to be well handled
Does preliminary evidence suggest that CommonGround works?		
Providing a space for shared understanding and support	Was there a reduction in feelings of isolation and loneliness and an enhanced sense of community? Did participants have enhanced access to spaces to share experiences and sense of commonality?	Mixed, some strong sense, others saw potential, others strongly felt that it was not possible Mixed, some felt it but several barriers emerged
Improved knowledge sharing	Did people improve their knowledge on the interaction between physical and mental health? Did people improve their self-management skills?	Mixed, some did and found useful tips, others felt engagement was too low, relied on other peer support Unclear, not explicitly asked during interview, many seem to have a good preestablished understanding

a ToC: Theory of Change.

#### How Could CommonGround Be Improved?

Throughout their interviews, many participants suggested areas for improving CommonGround (Table S1 in Multimedia Appendix 3 for the full results; discussed as appropriate earlier). Participants suggested improvements or adaptations to features with the view of enhancing ease of use and fostering a sense of community, which is critical if peer support is to flourish. Common suggestions included the removal of the 2FA process at login, creating a mobile app with push notifications, and subdividing the forum in some way to improve navigation. At the current time, CommonGround is not accessible to the general public.

## Discussion

### Principal Findings

This study evaluated the acceptability and usability of CommonGround, our online peer support and psychoeducation platform for people living with long-term conditions and subthreshold depression. By embedding qualitative analysis within a ToC framework, this study makes a methodological contribution to how complex digital mental health interventions can be designed and evaluated by demonstrating how feasibility-phase qualitative data can be used to evaluate assumptions and refine causal pathways. Through this process, we have identified lived experience–informed refinements for interventions (Table S1 in Multimedia Appendix 3) that enhance acceptability and usability and thereby maximize the opportunity to improve the mental health of the target population. Throughout the CommonGround project, the voices of lived experience experts have been central, including in the iterative process we undertook for producing the ToC. This underscores the importance of anchoring intervention design and associated ToC models in data from people with long-term conditions (or whatever the target audience may be) and co-investigators with lived experience.

Overall, this evaluation highlighted how CommonGround felt like a safe, credible, and comfortable space, with many valuing its potential to facilitate transdiagnostic social support and knowledge exchange. With 43 long-term conditions represented in the CommonGround community, this work addresses the lack of diagnostic diversity in research involving online peer support [19]. Limited time for organic growth, low volume of peer-generated content, and challenges in finding the time to engage were cited as reasons for the community not yet reaching its potential. Reviewing the rule of anonymity and improving the navigation of CommonGround were suggested as opportunities to improve usability and bolster engagement.

### The Acceptability and Usability of CommonGround

Participants’ emphasis on safety, credibility, and trust aligns with established evidence that meaningful engagement in online peer spaces depends on perceptions of security and safety [36,37]. CommonGround was perceived as a safe space due to the reputable ownership (King’s College London), appropriate and successful moderation, and the protection of privacy and anonymity, reinforcing previous findings on the role of transparency and trustworthiness in fostering a sense of community and credibility in online peer support environments [38]. Many placed value on the fact that CommonGround had been created *for* people with long-term conditions *by* people with long-term conditions. This helped the space feel protected for those with chronic illness and aligned with their psychosocial needs, underscoring the benefits of co-producing interventions from the outset [39,40]. Anonymity was generally seen as enabling openness and vulnerability [41], although some felt it limited the depth of social connections that could form. While potential risks of anonymity (eg, trolling) were recognized [42], these were not experienced on CommonGround. This tension between anonymity and social connection is often noted among members of online communities [36,42], collectively suggesting that the rule of anonymity could be reviewed to accommodate personal preferences, especially in contexts where other factors, such as reputable moderation, may be sufficient to create a sense of safety independent of anonymity. Examining acceptability through the ToC enabled these findings to be interpreted as intervention mechanisms rather than isolated user preferences, providing actionable insights to refine the intervention. This illustrates the broader value of ToC for understanding and strengthening complex digital health interventions, alongside outlining critical aspects of acceptability that must generally be considered when designing digital interventions.

CommonGround was widely considered as simple, attractive, and intuitive to use, characteristics known to influence engagement with online platforms [43]. The extent to which CommonGround can satisfy complex accessibility needs, however, remains unclear and warrants further development (eg, voice-operated functionality) with lived experience experts who have additional needs. Future development work should also address a navigational barrier that affected engagement, notably the 2FA process at login. Although participants recognized the security benefits of 2FA [44,45], it often caused irritation and deterred logins, highlighting the need to balance safety with the ease of access while being mindful that personal preferences for security can vary, particularly among those who are skeptical of peer support or who are previous victims of cybercrime [46]. Other navigational challenges may also have contributed to the low engagement with the “resources page”, which were underused despite the psychoeducation being perceived as reliable and helpful by those who accessed them. As psychoeducation has been shown to improve self-management knowledge and self-efficacy [47-49], addressing any usability and navigation issues that limit engagement with them is critical. Participants’ frequent suggestion to develop a mobile app is consistent with evidence that apps can improve usability and engagement [47] and warrants exploration in co-design workshops and future evaluations. By evaluating intervention usability through the ToC, we were able to identify the specific intervention features that supported or hindered engagement and redefine what intermediate outcomes and indicators were most relevant to our target audience, rather than treating usability as a single construct. This demonstrates the broader value of iteratively refining the ToC through such evaluations to ensure acceptability and usability principles align with the needs of the target audience and to prioritize design modifications before large-scale evaluation.

For online communities to be successful, people also need to be willing to engage, share content, respond to others, and foster deep personal connections. On CommonGround, participants’ willingness to share was partly driven by their belief that peer support can provide mutual understanding and knowledge exchange, consistent with prior work noting how peer support facilitates sharing without a fear of judgment and feeling “seen” by people “like me” [36,42]. Notably, most participants were initially unsure of their opinion of peer support (see preprint [48] for full feasibility trial results), suggesting that initial open-mindedness to peer support rather than prior endorsement or experiences might be sufficient for uptake. It appeared that positive opinions of peer support were shaped through their interactions on CommonGround, highlighting the value of experiential exposure on CommonGround in forming beliefs of the value of peer support. This does, however, also highlight the need to promote awareness of peer support among the wider patient population too, as this term is often not used colloquially [49] and people may have negative preconceptions around the propensity for negative interactions, which could serve as a barrier to accessing peer support [19,50,51]. Although information about “what is peer support?” was shared on CommonGround, it could be beneficial to share this information during recruitment or onboarding to help manage expectations, facilitate engagement with the community, and tackle stigma about patient communities more broadly.

Despite general willingness to engage, overall participation in CommonGround was relatively low. By evaluating the intervention through a ToC lens, we were able to move beyond describing the low engagement to identify and refine which assumptions underpinning engagement were, and were not, realized in practice. For instance, the assumptions that appear unmet include participants’ limited time and fluctuating capacity to engage within the 12-week trial period. Engagement was also influenced by current personal needs, symptom fluctuations, and their stage in chronic illness trajectories [52], meaning that CommonGround was not always perceived as relevant at the time of the trial, although many anticipated future use given that fluctuating nature of mental-physical well-being. The mixed-diagnosis ethos may also have contributed to the low engagement: while many endorsed the transdiagnostic ethos, others expressed a preference for condition-specific content or hesitancy to share with those who may not understand the intricacies of their specific diagnosis. The fact that this is a novel approach versus the traditional condition-specific spaces people are accustomed to may mean the participants were hesitant to engage due to the unfamiliarity and concerns of whether this mixed-diagnosis community could “work” in reality. These findings reflect existing evidence that some self-management needs are diagnosis specific [53] and that the provision of peer support is no “one size fits all” [12]. Within the context of CommonGround, these findings suggest that engagement with CommonGround will vary over time, depending on both community culture and activity but also external personal factors. In a future trial, it is essential that the community is active for longer to maximize the opportunities for engagement, allow community norms to develop, and comprehensively assess the acceptability of the all-diagnosis-welcome approach. More broadly, these findings also highlight the importance of evaluating the assumptions that precede engagement with digital online health interventions to help facilitate their uptake.

### The Potential Benefits of CommonGround

Further evidence is needed to determine the impact of CommonGround on well-being, as we found a wide range of experiences relating to sense of community, sharing experiences, and a sense of commonality. Low engagement likely constrained the capacity for CommonGround to form the vibrant, self-sustaining, and active community that facilitates the benefits of peer support, particularly as a sense of community is a significant predictor of one’s contributions to online health spaces and the experience of positive outcomes [54]. Participants’ desire and motivation to post themselves may have been influenced by the low posting activity of others [55]. Moreover, it is common for the majority of participants in online communities to engage with a community silently without creating content themselves [56]. While participants did appreciate the discussion-stimulating posts of the engagement team, there was a clear desire for more content from their peers to improve their experiences, illustrating how there is a delicate balance of promoting versus stifling conversation when managing online communities [38,57]. Increasing the size of the community (ie, sample size of the trial), refining engagement strategies, improving navigation, and developing an app may collectively enhance acceptability and usability, thereby strengthening community culture and allowing for a more robust evaluation of CommonGround’s effectiveness in supporting the mental well-being and preventing the development of depression among those with long-term physical health conditions. This finding also reflects the broader challenge for the design and evaluation of interventions such as CommonGround, where intervention effectiveness is inherently interdependent, relying not only on participants engaging with the intervention themselves but also on the engagement of others to sustain the social processes through which benefit(s) may be achieved.

### Limitations

There are some limitations that should be noted when interpreting our findings. First, those who agreed to an interview are likely to have a bias toward higher levels of engagement with online peer support as they are proactive or interested in social support or self-management. Although we included participants who had various engagement levels to mitigate this, there might still be some perspectives that were not captured, particularly among those with a strong dislike or uncertainty about peer support. Second, our sample may also be biased in terms of digital literacy, access to the internet, and devices needed to engage. While digital literacy was not formally assessed, all participants would have had some degree of competency to take part in this entirely online study. Furthermore, having access to the internet was an inclusion criterion, and although it was not specified that this access must be in the home (ie, participants could exclusively access it via free public Wi-Fi points), there is the chance that limited internet access or access to private spaces to use the internet may have limited engagement with the intervention and research [58]. Although beyond the scope of the feasibility trial, considering ways to promote digital inclusion will be necessary in future trials to ensure inclusivity of those who may benefit from CommonGround. Finally, some interviews were conducted up to 6 weeks after the intervention finished. Some participants noted difficulties in recalling the specifics of CommonGround as they no longer had access to it and could get confused with other online platforms. In future work, screenshots of CommonGround could be provided if needed to prompt memory recall.

### Conclusions

CommonGround, our co-produced online peer support and psychoeducation platform, was valued for being a safe and credible space to connect with those with mutual understandings of long-term physical health conditions. Embedding qualitative evaluation within a ToC framework enabled systematic inquiry and refinement of assumptions, intermediate outcomes, and associated indicators underpinning the usability, acceptability, and potential benefit pathways of the intervention. Through this process, we also identified lived experience–informed development opportunities to enhance intervention design ahead of a future trial of CommonGround exploring clinical efficacy. The specific insights related to the CommonGround platform can inform the design and delivery of future online peer communities and highlight the assumptions and preconditions required to foster meaningful engagement and sustained participation. Beyond this, this work also demonstrates how embedding a ToC framework within intervention design and evaluation, and evaluating and refining components of the ToC, facilitates a more nuanced understanding of how and why digital (mental health) interventions may work, for whom, under what circumstances, and how interventions can be improved prior to widescale implementation.

## Supplementary material

10.2196/92384 Multimedia Appendix 1 Screenshots of intervention.

10.2196/92384 Multimedia Appendix 2 Topic guides.

10.2196/92384 Multimedia Appendix 3 Areas for CommonGround intervention optimization.

10.2196/92384 Checklist 1 SRQR checklist.

## Abbreviations

2FA: two-factor authentication
fRCT: feasibility randomized controlled trial
NHS: National Health Service
PHQ-8: 8-item Patient Health Questionnaire
RAG: Research Advisory Group
SRQR: Standards for Reporting Qualitative Research
ToC: Theory of Change
