# Supplementary material for: Improving CommonGround, an Online Peer Support Intervention for People Living With Long-Term Physical Health Conditions and Subthreshold Depression: Theory of Change–Informed Qualitative Study
Source: J Med Internet Res. 2026 Nov 3;28:e92384. doi: 10.2196/92384 (PMC13730410; doi:10.2196/92384)
Supplement: Supplementary file 1 [file jmir-v28-e92384-s001.docx]

## Appendix 2

**Screenshots of the final prototype of CommonGround**

Screenshot 1. The landing page of CommonGround

Screenshot 2. The community feed of CommonGround (peer support component)

Screenshot 3. The resources page of CommonGround (psychoeducation component)

Screenshot 4. The ‘My Garden’ page of CommonGround

Screenshot 5. Search box and search results page of CommonGround

Screenshot 6. Further support and crisis page

Screenshot 7. Moderation policy page

Screenshot 8. Admin panel
