# Supplementary material for: Improving CommonGround, an Online Peer Support Intervention for People Living With Long-Term Physical Health Conditions and Subthreshold Depression: Theory of Change–Informed Qualitative Study
Source: J Med Internet Res. 2026 Nov 3;28:e92384. doi: 10.2196/92384 (PMC13730410; doi:10.2196/92384)
Supplement: Supplementary file 2 [file jmir-v28-e92384-s002.docx]

# Appendix 3: Interview topic guides.

## [1] Control group topic guide.

*Introduction*

1. Why were you first interested in the CommonGround project?
2. Where do you usually go for information on managing your mental and physical health? Why do you choose these sources of information?
3. What does peer support mean to you? Is peer support something you have come across before?
4. Can you tell me about your experiences of participating in our research?
5. How did you hear about the study? How would you like to hear about something like this in future?

*Engagement*

We want to ask you what you thought about the NHS Mental Health webpages that we shared with you via email. (check in that it was not in their spam emails)

1. Did you use the NHS Mental Health webpages?
   a. If not, why not?
   b. If so, what pages did you explore?
2. Have you previously seen these NHS Mental Health webpages? If so, how did you find out about them? Have you ever been told about them by a healthcare professional?
3. Do you feel that these are relevant or useful to someone living with a long-term condition? If so, how? If not, what would make them relevant or useful (if anything)

How did you find the fortnightly emails from the research team? Do you remember receiving them? What did you think about the fact we shared the links via email to you?

a. How did you feel about the frequency of the emails?
b. Was there anything that you particularly liked or disliked about the emails we sent you?
c. How did these emails compare to other emails you may have or have not received with information about managing your LTC or mental health?

*Trial Features*

1. How did you find the ‘wait period’? As a reminder, the wait period happened after you told us you were interested, before we were ready to launch the trial and got you to complete your first questionnaire.
2. How did you find the length of the study overall? How did you find the length of the intervention period (i.e., the three months of email circulars of the NHS webpages)?
3. How did you find the questionnaires that we asked you to complete?
4. Throughout the research trial, was there anything that you found difficult or did not understand?

*Conclusion*

1. Is there anything you think could have been done differently in our study and what we asked you to do?
2. If a peer support platform (Like CommonGround where you could connect with other on a discussion forum and access evidence-based self-help information) was freely available in the future, where would you like to hear about it?
3. Overall, if you were to describe your experiences of participating in this study in 3 words what would they be?

## [2] Intervention group topic guide: Never signed up or never logged in.

*Introduction*

1. Why were you first interested in the Common Ground project?
2. Where do you usually go for information on managing your mental and physical health?
3. What does peer support mean to you? Is peer support something you have come across before?
4. Did you ever log into Common Ground? Did you use the platform?

*Engagement with CommonGround*

1. What would you say the main reason(s) for not using CommonGround were?
2. CommonGround included a library of evidence-based self-help information to help people with long-term conditions manage their physical and mental well-being. What do you think about these psycho-educational resources on CommonGround?
3. The peer support platform included an online discussion forum called the community page. You could create posts, ask questions, share your experiences, react and comment on other people’s posts. What do you think about this feature?
   a. If you did not use it, why not?
4. The My Garden space was a section where you could save things to a personal library. What do you think about this feature?
   a. If you did not use it why not?
5. The moderation team role was to make sure that people followed the Community Principles when using CommonGround. Their role involved reviewing, editing and deleting posts and guiding discussions were necessary.
   a. What were your experiences (if any) of the Moderation team during the trial?
6. The Engagement Team role was to encourage engagement through weekly emails and through posts, summarise trending discussions and shared relevant topics for people living with LTCs.
   a. What were your experiences (if any) of the Engagement Team during the trial (on the platform and via email)?

*Features and Functions of CommonGround*

1. If you did create a platform, how did you find creating an account on the platform?
2. What did you think about CommonGround being an anonymous platform?

*Trial Features*

1. How did you find the ‘wait period’? As a reminder, the wait period happened after you told us you were interested, before we were ready to launch the trial and got you to complete your first questionnaire.
2. How did you find the length of the study overall? How did you find the length of the intervention period (i.e., the three months of CommonGround access)?
3. How did you find the questionnaires that we asked you to complete?
4. Throughout the research trial, was there anything that you found difficult or did not understand?
5. How did you feel about CommonGround being a place for people with any physical health condition?

*Future of CommonGround and Closing*

1. Thinking about the future, what other features/functions would you like to see added to a platform such as CommonGround?
2. Is there anything you think could have been done differently in our study and what we asked you to do?
3. If CommonGround was freely available in the future, would you use it? Why or why not?
4. Who do you think would benefit most from CommonGround? Who do you think that CommonGround is designed for?
5. Reflecting on everything we have spoken about today; do you think being involved in the study helped you in any way? How?
6. Overall, if you were to describe your experiences of participating in this study in 3 words what would they be?

## [3] Intervention group topic guide: Platform users.

*Introduction*

1. Why were you first interested in the Common Ground project?
2. Where do you usually go for information on managing your mental and physical health? Why do you choose these sources of information?
3. What does peer support mean to you? Is peer support something you have come across before?
4. Did you ever log into Common Ground? Did you use the platform?

*Platform use*

1. What did you use CommonGround for? What motivated you to login?
2. The peer support platform included an online discussion forum called the community page. You could create posts, ask questions, share your experiences, react and comment on other people’s posts. What do you think about this feature? Did you use it?

**If not,** why not?

a. Prompts: Was it related to the topics and content? Was there something else you would like to see covered?
b. Was it related to how accessible or inaccessible the resources were? Your time? The reasons why you joined the platform?

**If you did:** What parts of this feature did you like? How did you use the community page?

3. CommonGround included a library of evidence-based self-help information to help people with long-term conditions manage their physical and mental well-being. What do you think about these resources on CommonGround? Did you access them?

**If did not use,** why did you choose not to look at the resources on CommonGround?

**If you did:** How did you use the resources and how did you decide what resources to look at?

4. The My Garden space was a section where you could save things to a personal library. What do you think about this feature?

**If not,** why not?

**If you did:** how and why did you use it?

5. When and how did you use CommonGround?
6. What did you like about using CommonGround?
7. Was there anything you did not like about using CommonGround?

*Features and Functions*

1. How did you find creating an account on the platform?
2. What did you think about CommonGround being an anonymous platform?
3. What were your experiences (if any) of the Moderation Team on CommonGround? What effect did they have on CommonGround? Tell us about any noticeable interactions you had or observed.
4. What were your experiences (if any) of the Engagement Team during the trial (on the platform and via email)?
5. Could you tell the difference between the engagement and moderation Team?

*Peer support and suitability*

1. Did you feel that a community was formed on CommonGround?
2. How did you feel about CommonGround being a place for people with any Long-term health condition?

**Trial Features**

1. How did you find the questionnaires that we asked you to complete?
2. Throughout the research trial, was there anything that you found difficult or did not understand?
3. How did you find the length of the study overall? How did you find the length of the intervention period (i.e., the three months of CommonGround access)?
4. How did you find the ‘wait period’? As a reminder, the wait period happened after you told us you were interested, before we were ready to launch the trial and got you to complete your first questionnaire.

*Future of CommonGround and Closing*

1. Thinking about the future, what other features/functions would you like to see added to a platform such as CommonGround?
2. Who do you think would benefit most from CommonGround? Who do you think that CommonGround is designed for?
3. If CommonGround was freely available in the future, would you use it? Why or why not?
4. Reflecting on everything we have spoken about today; do you think accessing CommonGround helped you in any way? How?
5. Overall, if you were to describe your experiences of participating in this study in 3 words what would they be?
