# Supplementary material for: Improving CommonGround, an Online Peer Support Intervention for People Living With Long-Term Physical Health Conditions and Subthreshold Depression: Theory of Change–Informed Qualitative Study
Source: J Med Internet Res. 2026 Nov 3;28:e92384. doi: 10.2196/92384 (PMC13730410; doi:10.2196/92384)
Supplement: Supplementary file 3 [file jmir-v28-e92384-s003.docx]

# Appendix 3. Areas for CommonGround intervention optimisation.

Table S1. Potential changes to CommonGround that could be implemented based on participant experiences.

| **CHANGES TO INTERVENTION** | | |
| --- | --- | --- |
| **Area of platform** | **Quotes** | **Suggested changes or improvements** |
| 2FA and login | **Participant 11, Content creator:** “The only thing that might make it better is maybe using the way you log in to your device, instead of using a text, like your fingerprint, or a pattern, or your PIN code for getting in. That, I get. That might be a better idea, especially if people are logging in on a laptop and then have to go and find their phone out to get a text. But that's the only itch I've got with it.” | Make changes to the 2FA process, so that it is less frequent.  Potential to explore other options, such as personal PIN entered after email and password |
| Navigation | **Participant 6, Acknowledger:** “I wondered, sometimes, about linking that into the forum itself. Like how you might have a general chat, you might have a weekly topic by someone posting up, oh, this week, I'm doing these gentle exercises, does anyone want to do them with me? That could help people… Because I think, sometimes, it's focusing your mind to the right bit of information.” | Better linkage between the community feed and resources page. For instance, to be able to link to a psychoeducation resource within your post in the community feed |
|  | **Participant 22, Content creator:** “I think there could have been a little bit more organisation in terms of topics. In some ways having broader topics at the top, so personal, financial job worries, health. Particular health issues or particular health diagnoses, if there were subsections for those, I think that might have helped organise it a little. Because at times I'd look and see there are about 20 different topics here. I don't know if I'm going to look through all of them.” | Divide up the topics of the psychoeducation resources into a smaller set of broader topics, with subtopics within them. |
|  | **Participant 43, Reader:** “Maybe the layout might have been a bit better as well, compared to how it looked for me. Obviously, I've just found out that I've missed out all the resources and things, so maybe an app would have been clearer on what information was actually there, as well.” | Review layout and navigation of the platform sections and features, conducting some usability testing to identify the specific edits to be made |
| Accessibility | **Participant 27, Content creator:** “though my speech is not great, it allowed you to use microphone input device. I think that might be useful. […] I think that was another reason I didn’t participate as much as I would like to. Is the fact that the typing is difficult for me to type, even though I can. I think I can’t be bothered to write a lot.” | Implement voice-to-text options for creating post and creating comments |
| Community feed | **Participant 6, Acknowledger:** “Maybe if it were to be split up, split up more towards different experiences. So, for people who are stuck in bed every day or housebound, their life experiences and their needs, or frequent hospital flyers, if you had a board which was frequent hospital flyers, or stuck at homing, or working.” | Divide the community feed into multiple different feeds, for instance, in relation to life/different experiences (i.e., not necessarily condition specific) |
|  | **Participant 8, Acknowledger:** “It would help to have a filter button on it, you filter down, but not the condition.”  **Participant 16, Acknowledger:** “I think maybe a filter of some kind, potentially on topics, or maybe selecting keywords from some of the conversations. Something like mental health or pain, or some kind of filterable thing of common keywords that are used might have been good. Or even a date filter, because I knew when I'd been on before, so I’d knew what I'd seen already. So if it was most recent or today's posts, that's useful, too. Any kinds of those filters would have helped me, I think.” | Potentially move the existing search tool from the navigation bar to be embedded at top of the community feed |
|  | **Participant 30, Content creator:** “I think the majority of people who've had access to a specific group will want to do that, but won't be disinterested in the bigger picture.”  **Participant 31, Content creator:** “So just it would be really helpful to have specific forums for conditions. It was interesting to hear about what other people were going through. I think that's great as well. But I would have really benefited from maybe a forum just for people with IBD or Crohn's and colitis. Or people that have recently been diagnosed. Or maybe even giving people the option to create forums, depending on what they see as a need.” | Options to create subcommunities if members wish to |
| Psychoeducation resources page | **Participant 6, Acknowledger:** “Potentially just a news thing for research and interesting new things coming in. […] I think it could be interesting as its own separate tab, for the people who want a bit of reading, but they don't want to… Not necessarily a self-help. Just something just to get their teeth into”  **Participant 19, Acknowledger:** “Maybe more resources that are condition specific” | Expand current repertoire of topics, including condition-specific features  and new research / clinical guidance |
|  | **Participant 41, Reader:** “Sometimes the thought is to put as much content in as possible, which then makes it more difficult to navigate. Rather than having core things and then maybe building on that as people say, could we have a little bit about this, could we have a little bit about that? Rather than trying to be all things to all people to start off with.” | Build the resources page as the community evolves in relation to the wants of the community |
|  | **Participant 16, Acknowledger:** “It would be good to have a method of it sharing to the resources section on the app as well, so that anyone else can reference it. Because again, you don't want to be searching posts.” | Potential for peer-sourced information to be reviewed and integrated into the psychoeducation page |
|  | **Participant 31, Content creator:** “that would be nice, it just popped into my head, to have a central place where if you have this condition or that condition there are these support groups. Not everybody is aware of the different support groups in their area.” | Include information on local self-help groups that people might wish to attend |
|  | **Participant 29:, Content creator** “So, I’m fine with reading lots of text in a PDF or whatever. Other people would prefer, maybe, infographics, or video, or whatever that might be. So, having that variety, I suppose, to meet different people's needs would be useful, too” | Review the format of resources, and potentially include resources in audio or visual formats |
| Moderation | **Participant 6, Acknowledger:** “Possibly having a system where you couldn't do things like that [upload attachments/post without review by moderators] unless you'd been a member for a certain amount of time, and been active, because that stops people joining easily for the pure motive of grooming or trying to take advantage of other people.” | Features/functions become available in a staged-approach dependent on length of time they have been a member. For instance, post-approvals initially required, ability to upload attachments. |
|  | **Participant 6, Acknowledger:** “if possible, having roles of peer support for peer support mentors or something, or moderators, whatever is the right term or the right setting for people who are longer-term diagnosed, because their knowledge is useful. And they can help redirect a topic, I think, for newly diagnosed, which is going in the wrong direction, where they might not feel confident to join in otherwise, because they're not newly diagnosed. But they have the experience to go, actually, I can feel this is getting a bit competitive right now, or this is getting a bit… Because it does. And I do think it's human nature, but there are some weird dynamics of these communities, sometimes, and it’s trying to keep them grounded.” | Explore the potential to have peer moderators involved |
| Engagement | **Participant 15, Reader:** “But I think it just gave a slightly different flavour, I guess. And so maybe, having those separated out somehow. I think they were pinned when they posted. I think they were at the top, or I think a pretty colour, or just at the top. I don't know if something could be, like they appear differently, or a different channel or something, because it was just a slightly different vibe than just like general chat.” | Adjust the pinning of engagement posts |
| Fix glitches | - **Participant 43, Reader:** “And so, I tried to upload an image from one of my favourite quotes, but it wouldn't let me do so.” - **Participant 32, Never logged in:** [interviewer: So, you had some problems with that logging in code?] “Yes. When you have difficulty like that you tend not to, well, after a while you just think, ugh.” - **Participant 44, Reader:** “I think I went to [save posts to My Garden private page], but I think I had a problem with doing so. So, I think I went to save something there, and it didn't quite work for me, for whatever reason. So, I gave up on it, to be honest. So, I liked the idea of that thing of saving what you needed to, in a space to save it in, if you wanted to. But, as I say, it didn't quite work for me.” - **Participant 11, Content creator:** “I'd save things there, or thought I'd saved things there, but then. So, when I’d go back to it, those things weren't there, and I'd have to search again, and then they would come up, but I definitely had saved them to My Garden, but it hadn't saved what I expected it to save.” - **Participant 9, Content creator: “**Sometimes I went in the community page on discussion, I came out of it, it took me to the top of the page and take me back to where I was. That was a problem.” - **Participant 12, Content creator:** “When I went on to see it, my notifications told me people responded. If I clicked on them, it would not show me them. And that was the case for, I think, at least couple of days. So, it wasn't just on the day.” - **Participant 12, Content creator:** “There were things you could add. But things like your profile, and if you added them in and saved them, it didn't save again. The glitchy thing that I never raised.” | Fix glitches in:   - Image upload - 2FA codes being received - Posts saving to My Garden - Glitchy scroll - Notification system - Saving updates to the profile page |
| Create an app | **Participant 6, Acknowledger:** “Sometimes having an app rather than a website is easier, just because you can put it on your homepage on your phone, and it's easier to remember it exists. And just might get push notifications that come up, and you can stay logged in”  **Participant 16, Acknowledger:** “if I had this as an app on my phone, I would use it in exactly the same way as social media or any kind of resource platform” […] **“**I think definitely if it was launched as an app, that would be amazing.”  **Participant 34, Never signed up: “**And then having access to an app or something a bit more mobile-friendly would be great.” | Addition to the web-based version, create an app with push notifications |
| Updates to existing features | **Participant 12, Content creator:** “if there was a proper emoji, and they were seeing that people were putting on that emoji, caring for them, or whatever, then they could say, well, I've got support here, even though there's nothing been said.” | Review and expand the range of emojis, considering those that are typical of social media sites |
|  | **Participant 15, Acknowledger:** “There probably had to be a reason we were trying to tag each other. It wasn't so easy to use, because you had to get that exactly right.” | Improve tagging feature, so that when you begin to type username suggestions automatically appear |
|  | **Participant 16, Acknowledger:** “Or you're logged in for a certain amount of time and it will auto log you out if you haven't used it in three days, for example. I think that would be useful. Someone like me, I'd prefer to leave it logged in if I was using it a lot. But I think for most people, it's good practice to log in and out of things anyway, because people forget that for security reasons, so I would always encourage that. An auto log out might be the best option after a period of time.” | Adjust auto-logout after more prolonged periods of inactivity |
|  | **Participant 11, Content creator:** “So I then couldn't find post that people had done, that I'd commented on. I'd have to go in and search for that. So it might be that one of the options is a menu option that says, posts you’ve commented on, something like that, so that you know… Basically, it's your history, and that option didn’t appear to be there.” | Expand ‘recent activity’ to include posts that someone has commented on, in addition to their own posts |
| New features that could be created | **Participant 31, Content creator:** “think it would be a good idea to have your own symptom tracker on there, like your own mental health symptom tracker, that's unique to you, that no one else sees unless you want to share it. Because I think that would be quite useful for you to track how you're doing, not necessarily for everyone else to see, but you to see how you’re getting on.” […] “It would be great to have a section where you can add notes as well […] because I feel like I've got notes stored everywhere. It would be nice if it was actually stored in the platform as well.” | Private space to track symptoms and/or to add notes or keep a diary |
|  | **Participant 12, Content creator: “**But if you're already in the platform, maybe something being like, now start a conversation about, and a suggestion, maybe that would be helpful. Rather than go into that platform thinking from scratch. So maybe make it even easier for people to talk about just ideas for stuff to talk about.”  **Participant 31, Content creator:** “One of the things, it's actually quite a nice thing if you’ve logged in, one of the things that pop up straight away would be, I am feeling good today, so-so, not too good, I'm feeling down. And you could post that. And then you’d get people who are in your group, because, again, you could form your own group, and you’d get people who said, oh, I'm sorry to hear that” | Automatic prompts to start discussions, which could be topic suggestions, or simply rating ‘how do I feel today’ on a scale, which becomes a post in itself |
|  | **Participant 16, Acknowledger:** “just to have something under the avatar or something just to say, Interviewee is offline for this period. Something, yes. It's a good shout.” | Build some feature to illustrate that someone is away from CommonGround for a prolonged period |
|  | **Participant 19, Acknowledger:** “I think if you could see the numbers who had the same condition or something or just know that there were people there who might get it, because I guess everyone doesn't want to share all the stuff. But like a high-level summary of how many people with different conditions and stuff there are. Just so you know if they might get it, because I guess you don't want to post things and then have no replies, because that's really sad. […] just have a summary of who else is there, what conditions they have. Just, I don’t know, a high-level chart or something that shows the number of people with different conditions or something, just so you can get a sense of who else is there. | Create an overview of who the community members are, and who is online at that time |
|  | **Participant 41, Reader:** “You could do, like you say, a voice note. Again, people might not like the voice note because they worry about how they sound on it. But voice notes will work. You can maybe have an opportunity to do your voice note” | Option to add a voice note as a post and/or a comment |
|  | **Participant 39, Never logged in:** “you can turn off comments at some point because you're [unclear] having notifications. Otherwise, you become obsessed, and it takes over your life.” | Option to turn off comments on your own post |
|  | **Participant 22, Content creator:** “I would have said, perhaps, as I say, breaking it down into topics and then saying, there's been a lot of activity in our topics about a particular subject. Or lots of people are interacting with this thread.” | Highlight recently active threads / threads that have had a lot of activity |
| New feature: Optional private messaging | **Participant 16, Acknowledger:**  Participant: “maybe could have had the option to, if you want to contact this person to speak further, then yes, because there may have been someone on there that had exactly my illnesses and exactly the same point in life, and I might have wanted to chat to them separately on a private message”  Interviewer: “Private message within the CommonGround platform, yes?”  Participant: “Yes. Within the CommonGround, yes. I feel that would be safer, but then it's your choice if you go elsewhere with that. But within the platform is a good option, yes.”  **Participant 38, Never logged in:** “Having chat function would be nice. Bit of a swap experiences and what have you.” | Create a private chat function, which may involve:  [1] Request to connect via private chat that must be accepted by both members  [2] Moderator access to chats to review content, and option to report chat |
| Who can join | **Participant 21, Content creator:** “It's very difficult to live with someone with a long-term condition, and they need just as much support as the person with the condition. Yes, that would be brilliant if CommonGround opened up to that. […] Or, if you're happy, if you and your daughter, say, want to do a post together, you could do that. And that may then encourage other people to do that. But I think a lot of these things, very often, it's, me, me. And it's not just me. It's everyone in my family.”  **Participant 43, Reader:** “So, I think it'd be really crucial to include significant others, if you like, given the opportunity, if they want to comment, and we even just have a section for them as well, you know?” | Explore whether more people share the view that the space should be opened to family/carers, and if so, whether this should be within the main community or a subcommunity |
| **CHANGES TO TRIAL** | | |
| **Area of trial design** | **Quotes** | **Suggested changes or improvements** |
| Intervention period | **Participant 31, Content creator:** “I would like access to that platform for the rest of my life, if possible. I think it's fantastic. As part of the study, I think that, three months, it’s a bit short […] I think a bit longer might have been better, so maybe six months, nine months, maybe even 12 months, because a lot can change in a year, mental health-wise”  **Participant 19, Acknowledger:** “maybe that’s not ideal, because I think it takes a while for people to get comfortable to start posting and for the conversations to start flowing. So, I think maybe a bit longer would be good” | Increase the length of the intervention period to account for [1] growth of a community [2] fluctuating ability to engage dependent on life events |
| Closure of platform after the intervention period | **Participant 15, Reader:** “I found it hard that you couldn't access the platform once it had closed.”  **Participant 11, Content creator:** “it's frustrating that I've been able to take part in that research and found it very helpful, and now it's gone, and I'd like to be able to access it again, and to make use of it.” | Consider whether the platform must be closed at the end of the intervention period |
